# An early increase in endothelial protein C receptor is associated with excess mortality in pneumococcal pneumonia with septic shock in the ICU

**DOI:** 10.1186/s13054-018-2179-6

**Published:** 2018-10-05

**Authors:** Agnès Chapelet, Yohann Foucher, Nathalie Gérard, Christophe Rousseau, Olivier Zambon, Cédric Bretonnière, Jean-Paul Mira, Béatrice Charreau, Christophe Guitton

**Affiliations:** 1 Medical Intensive Care Unit, Nantes University Hospital, Nantes, France; 2 Centre for Research in Transplantation and Immunology (CRTI) UMR1064, INSERM, Nantes University, Nantes, France; 3 Institute of Transplantation Urology Nephrology (ITUN), Nantes University Hospital, Nantes, France; 4 INSERM, UMR 1246 - SPHERE, Nantes University, Nantes University Hospital, Nantes, France; 5 Institut Cochin, INSERM U1016, Paris, France; 6 Medical Intensive Care Unit, Cochin University Hospital, Assistance Publique–Hôpitaux de Paris (APHP), Paris, France; 7 Medical and Surgical Intensive Care Unit, Le Mans Hospital, Le Mans, France

**Keywords:** Sepsis, Soluble EPCR, In-hospital mortality, Predictive score, Early biomarker, Sepsis outcome, Pneumococcal pneumonia

## Abstract

**Background:**

This study investigated changes in plasma level of soluble endothelial protein C receptor (sEPCR) in association with outcome in patients with septic shock. We explored sEPCR for early sepsis prognosis assessment and constructed a scoring system based on clinical and biological data, in order to discriminate between surviving at hospital discharge and non-surviving patients.

**Methods:**

Clinical data and samples were extracted from the prospective “STREPTOGENE” cohort.

We enrolled 278 patients, from 50 intensive care units (ICUs), with septic shock caused by pneumococcal pneumonia. Patients were divided into survivors (*n* = 194) and non-survivors (*n* = 84) based on in-hospital mortality. Soluble EPCR plasma levels were quantified at day 1 (D1) and day 2 (D2) by ELISA. The *EPCR* gene *A*3 haplotype was determined. Patients were followed up until hospital discharge. Univariate and multivariate analyses were performed. A scoring system was constructed using least absolute shrinkage and selection operator (lasso) logistic regression for selecting predictive variables.

**Results:**

In-hospital mortality was 30.2% (*n* = 84). Plasma sEPCR level was significantly higher at D1 and D2 in non-surviving patients compared to patients surviving to hospital discharge (*p* = 0.0447 and 0.0047, respectively). Early increase in sEPCR at D2 was found in non-survivors while a decrease was observed in the survival group (*p* = 0.0268). *EPCR A3* polymorphism was not associated with mortality. Baseline sEPCR level and its variation from D1 to D2 were independent predictors of in-hospital mortality. The scoring system including sEPCR predicted mortality with an AUC of 0.75.

**Conclusions:**

Our findings confirm that high plasma sEPCR and its increase at D2 are associated with poor outcome in sepsis and thus we propose sEPCR as a key player in the pathogenesis of sepsis and as a potential biomarker of sepsis outcome.

**Electronic supplementary material:**

The online version of this article (10.1186/s13054-018-2179-6) contains supplementary material, which is available to authorized users.

## Background

Severe sepsis is a frequent and serious disease in intensive care units (ICUs). It remains a leading cause of death in critically ill patients, despite efforts to improve patient outcomes [1]. Septic shock is associated with systemic inflammation and an exacerbated procoagulant state mediated by the tissue factor (TF) pathway. The protein C (PC) anticoagulant pathway is a major system that prevents coagulation, and its impairment influences outcome in sepsis. This pathway involves two soluble proteins: PC and protein S, and two endothelial receptors: thrombomodulin (TM) and the endothelial protein C receptor (EPCR). EPCR (CD201) is a 46-kDa type I transmembrane protein that is expressed mainly on the luminal surface of endothelial cells from large vessels and which is homologous to major histocompatibility complex class I/CD1 family proteins [2–4]. PC binding to membrane EPCR (mEPCR) increases PC activation by thrombin-TM complexes [3]. Activated protein C (APC) has anticoagulant effects enhanced by EPCR, but also exhibits anti-inflammatory effects via the protease-activated receptor 1 (PAR 1) [5, 6], and anti-apoptotic effects [7, 8] requiring binding to mEPCR in lipid rafts. Membrane EPCR also binds with factor VII on endothelial cells [9]. Disruption of the *EPCR* gene in mice causes placental thrombosis and embryonic lethality, confirming a key role for EPCR in controlling coagulation [10].

Soluble EPCR (sEPCR) levels have been described in human plasma during sepsis [11]. Soluble EPCR is generated by ectodomain shedding [12] mediated by TACE/ADAM17 [13] and/or by alternative messenger RNA (mRNA) splicing in haplotype-A3–carrying endothelial cells [14]. Soluble EPCR binds both PC and APC with an affinity similar to mEPCR [15]. While the role of mEPCR is clearly anti-thrombotic and anti-inflammatory, the function of the circulating sEPCR remains unclear. The binding of APC to sEPCR interferes with binding of APC to phospholipids and hinders factor Va inactivation. Furthermore, binding of PC to sEPCR enhances APC generation, suggesting a pro-coagulant effect of sEPCR [16]. Otherwise, in inflammatory diseases, such as systemic lupus erythematosus, the release of sEPCR contributes to renal lesions [17].

In a preliminary study, we reported that sEPCR plasma level was stable in a cohort of 40 ICU patients with severe sepsis [18]. However, we found that patients with a poor outcome at D28 had an early significant increase in sEPCR level at D2 post admission. This finding suggested that a rise in sEPCR level at D2 might be correlated with a poor prognosis and that sEPCR could provide an early biological marker of sepsis outcome. Our findings also strongly suggested an implication of elevated sEPCR in the pathogenesis of severe sepsis. The present study was conducted firstly to further evaluate the relationship between quantitative change in sEPCR level and mortality in a large and homogeneous cohort of patients with septic shock. Our experiments included the quantification of plasmatic sEPCR, at D1 and D2, together with the determination of *EPCR* genotypes in septic patients. We hypothesized that patients with early increase in sEPCR within 48 h of hospital admission would have a higher risk of in-hospital death compared to patients without such an increase. The secondary aim was to propose a prognostic scoring system for patients admitted to the ICU for septic shock, based on both clinical and biological variables, in order to discriminate between survivors at hospital discharge and non-survivors.

## Methods

### Study population

**Fig. 1:**
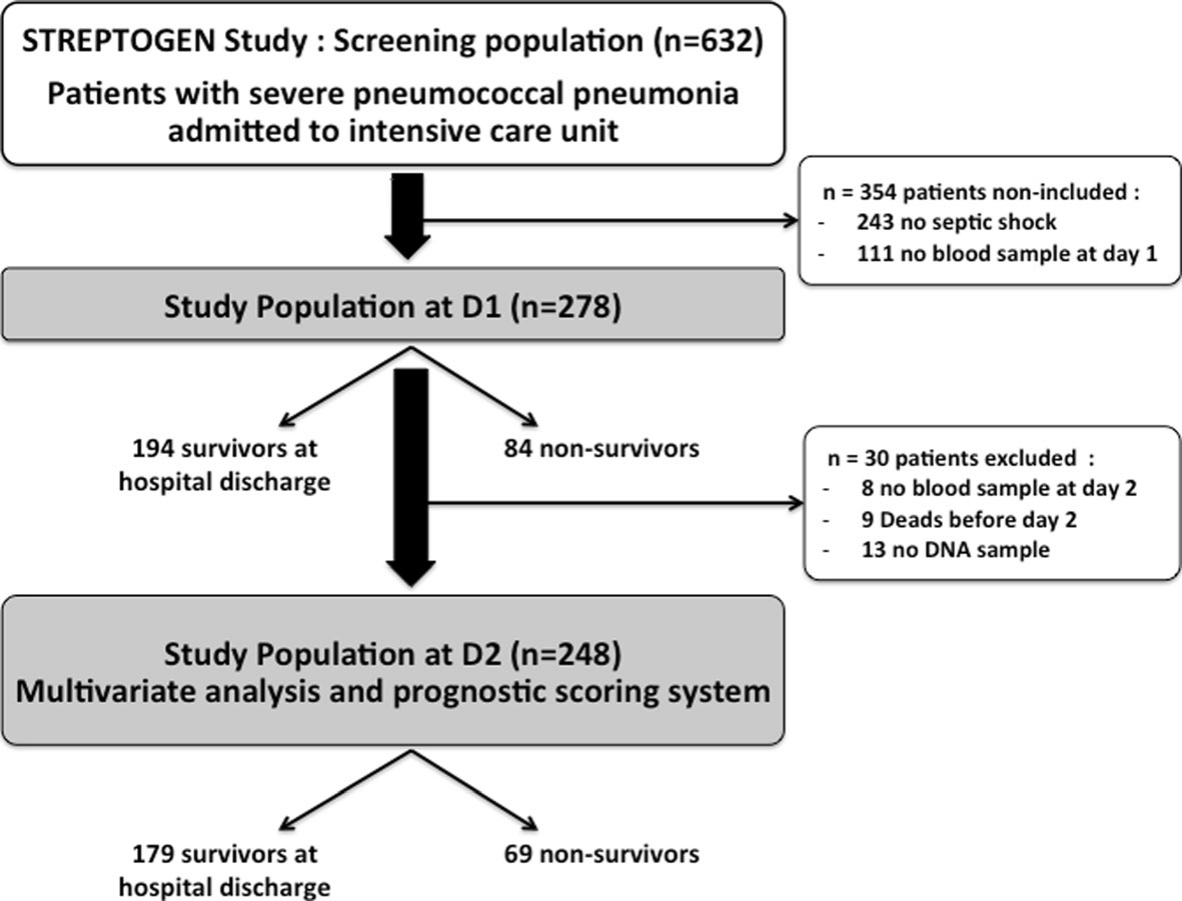
Study flow chart. D1, day 1; D2, day 2

Data and biologic samples were extracted from the prospective STREPTOGENE cohort, a multicentric prospective observational French study conducted in ICUs from 50 hospitals in France. Between December 2008 and February 2012, 632 patients with severe pneumococcal pneumonia and admitted to the intensive care unit were included in the STREPTOGENE study. Patients had no previous risk factors for pneumonia. Septic shock was defined as the need of norepinephrine during the initial care. Within this cohort, 389 patients had septic shock. Among them, 111 (28%) were excluded due to lack of blood samples at D1. The initial cohort included 278 patients (Fig. 1). For all patients, plasma sEPCR at D1 was measured by ELISA. We secondarily excluded patients without blood samples at D2 or without DNA samples. A total of 9 patients died before D2, blood samples at D2 were missing in 8 patients and DNA samples were missing in 19 patients. Among the 278 patients initially included in our study, 248 patients surviving at D2 were included in the final analysis.

Baseline characteristics of the patients were collected, including demographic information, severity score at admission, Simplified Acute Physiology Score (SAPS) II, Sepsis-related Organ Failure Assessment (SOFA), and comorbidities. DNA and blood samples at D1 and D2 collected and stored for the STEPTOGENE study were used in this ancillary study.

### Quantification of plasma sEPCR by ELISA

Blood samples were collected on admission to ICU (D1) and on the following day (D2). Samples were centrifuged at 3500 rpm for 10 min at room temperature and stored at − 80 °C until use. Quantification of sEPCR in plasma was performed using ELISA kits (Asserachrom, Stago-Diagnostica, France) according to the manufacturer’s recommendations. All assays were performed in duplicate.

### DNA isolation and *EPCR* genotyping

*EPCR* genotyping was determined for all patients to identify the occurrence of the *EPCR A3* allele, previously reported to affect baseline sEPCR plasma level [16]. Genomic DNA was purified from blood using PLC MagnaPure Compact (Roche Diagnostics, Meylan, France), diluted at a final concentration of 5 ng/mL, and stored at − 80 °C. *PROCR* gene polymorphism was determined, by allelic discrimination using quantitative PCR. The *EPCR* gene was screened using probes (rs867186, Life technologies, Carlsbad, CA, USA) targeting the single nucleotide polymorphism 6936 (A/G), characterizing the A3 allele of the *EPCR* gene. Sequencing was performed on Abi7900 (Applied Biosystems Applied Biosystems, Foster City, CA USA). The analyses were performed using SDS 2.4™ software. Allelic frequencies were calculated and expressed as percentages of patients carrying at least one *EPCR* A3 allele.

### Research ethics

The protocol of the study was approved by the institutional medical research review board of Saint Louis Hospital, Paris, France, department of Health and Human development and by the Commission Nationale Informatique et Liberté (CNIL). Written informed consent was obtained from the patient or surrogate, before inclusion, for the collection and storage of blood, cytokine assays, isolation of DNA and determination of gene polymorphisms. All data were anonymised. Tubes with anonymous barcodes were used for DNA collection (ABgene, Life technologies).

### Statistical analyses

The means ± standard deviation (SD) were calculated for continuous variables with a normal distribution. The number of patients in each category and the corresponding percentages were detailed for categorical variables. Crude comparisons were performed using the unpaired and two-sided Student *t* test for continuous variables, and chi-square statistics for categorical ones.

The principal endpoint was the in-hospital mortality. To construct the corresponding prognostic scoring systems, clinical variables or biomarkers levels were selected using least absolute shrinkage and selection operator (lasso) logistic regression [19]. All the variables were normalized. The following variables were defined as possible candidates: gender, septicaemia, body mass index, McCabe scale, A3 phenotype, age, Fine’s score, SOFA score, and sEPCR level at D1 and D2. The tuning parameter, i.e. the number of selected explicative variables, was estimated by maximising the 10-fold cross-validation criteria. An advantage of this method is to avoid the selection of the explicative variables by using the *p* value, which does not constitute a relevant indicator of prognostic capacities [20, 21]. The prognostic score was the sum of the product between the regression coefficients and the explicative variables, i.e. the linear predictor of the lasso logistic regression. The 0.632+ bootstrap estimator of the corresponding ROC curve was used in order to avoid over-fitting and for internal validation, as we previously proposed [22]. Statistical analyses were performed using R version 3.0.1 [23]. The package penalized was used for the lasso regression and the package *ROC632* for the bootstrap 0.632+ estimations.

## Results

### Description of the cohort

Demographic and clinical characteristics of the cohort at D1 (*n* = 278) are summarized in Table 1 according to in-hospital mortality. The ICU mortality was 28.0% (*n* = 78) and in-hospital mortality was 30.2% (*n* = 84). Mean age was 64.4 ± 15.5 years, and 60.8% were male. The mean admission SAPS II score was 52.9 ± 17.7 and the mean SOFA score at D1 was 9.8 ± 3.7, the mean Fine score was 143.9 ± 41.5, and the mean duration of ICU stay was 18.5 ± 19.14 days. As expected, many differences between non-survivors and survivors at hospital discharge have to be underlined. In particular, patients that died before hospital discharge were older and had significant higher mortality and morbidity scores.

**Table 1:** Population characteristics at day 1 (*n* = 278)

Population characteristics at day 1 (*n* = 278)	Cohort *n* = 278	Non-survivors *n* = 84	Survivors *n* = 194	*P* value
Patient age (years)	64.4 ± 15.5	70.1 ± 14.5	61.9 ± 15.3	<0.0001
Male, *n* (%)	169 (60.8)	62 (73.8)	107 (55.2)	0.003
Body mass index (kg/m^2^)^a^	25.3 ± 5.8	25.2 ± 5.2	25.4 ± 6.1	0.791
McCabe score at day 1, *n* (%)	252 (90.7)	72 (85.7)	180 (92.8)	0.063
History of:				
Cardiac disease, *n* (%)	138 (49.6)	53 (63.1)	85 (43.8)	0.003
Respiratory disease, *n* (%)	154 (55.4)	44 (52.4)	110 (56.7)	0.506
Diabetes mellitus, *n* (%)	58 (20.9)	22 (23.8)	36 (18.6)	0.150
Renal disease, *n* (%)	17 (6.1)	9 (10.7)	8 (4.1)	0.035
Immunologic disease, *n* (%)	34 (12.2)	9 (10.7)	25 (12.9)	0.612
Fine’s score	143.9 ± 41.5	163.9 ± 35.3	135.3 ± 41.1	<0.0001
SOFA score day 1	9.8 ± 3.7	11.6 ± 4.1	9.1 ± 3.2	<0.0001
SAPS II	52.9 ± 17.7	62.6 ± 17.2	48.7 ± 16.3	<0.0001
Norepinephrine at admission, *n* (%)	207 (74.5)	65 (77.4)	142 (73.2)	0.462
Mechanical ventilation day 1, *n* (%)	199 (71.6)	71 (84 .5)	128 (65.9)	0.002
Renal replacement therapy day 1, *n* (%)	30 (10.8)	21 (25.0)	9 (4.6)	<0.0001
Septicaemia, *n* (%)	117 (42.1)	38 (45.2)	79 (40.7)	0.484
Hydrocortisone, *n* (%)	167 (60.1)	59 (70.2)	108 (55.7)	0.023
Drotrecogin, *n* (%)	41 (14.8)	17 (20.2)	24 (12.4)	0.089
Length of stay (days)				
ICU	18.5 ± 19.14	16.7 ± 23.0	19.3 ± 17.2	0.298
Hospital	28.9 ± 26.3	18.7 ± 25.8	33.5 ± 25.3	<0.0001

Description of the population according to the status of patients at hospital discharge: survivors (*n* = 194) or non-survivors (*n* = 84). The mean ± standard deviation was reported for continuous variables. The number of patients in each category and the corresponding percentages are given for categorical variables. *P* values were obtained using chi square statistics for qualitative variables and the unpaired and two-sided Student’s *t* test for continuous ones

*SOFA* Sepsis-related Organ Failure Assessment, *SAPS* Simplified Acute Physiology Score

^a^19 values were missing for body mass index

### Time course of sEPCR levels at the onset of sepsis, and influence of *EPCR* genotype

sEPCR levels and *EPCR* genetic analysis are reported in Table 2. Plasma sEPCR levels at D1 were significantly higher in patients who died before hospital discharge compared to patients alive at hospital discharge (mean sEPCR = 102.5 ± 57.9 ng/mL vs 87.5 ± 53.0 ng/mL respectively, *p* = 0.0447). Similarly, mean sEPCR at D2 was also higher in the non-survivor group (108.8 ± 63.9 ng/mL vs 84.6 ± 50.1 ng/mL, *p* = 0.0047). The time course of sEPCR levels between D1 and D2 further revealed significant differences between the two groups. Indeed, an increase in the mean of sEPCR from D1 to D2 was observed in deceased patients (D2–D1 mean value 4.6 ± 26.9 ng/mL) while, in contrast, a decrease in patients alive at hospital discharge was found (D2–D1 mean value − 3.5 ± 23.1 ng/mL, *p* = 0.0268).

**Table 2:** sEPCR levels and polymorphism (n = 278)

	Global *n* = 278	Non-survivors *n* = 84	Survivors *n* = 194	*P* value
sEPCR level in plasma (ng/mL)				
Day 1, *n* = 278	92.0 ± 54.9	102.5 ± 57.9	87.5 ± 53.0	0.0447
Day 2, *n* = 261 ^a^	91.3 ± 55.2	108.8 ± 63.9	84.6 ± 50.1	0.0047
Delta EPCR: day 2 - day 1	−1.3 ± 24.4	4.6 ± 26.9	−3.5 ± 23.1	0.0268
*EPCR A3* haplotype, *n* (%) (*n* = 259^b^)	38 (14.7)	10 (12.8)	28 (15.5)	0.5804

Description of soluble endothelial protein C receptor (sEPCR) levels at day 1, day 2, kinetics between day 1 and day 2, and polymorphism *EPCR A3* in survivors and non-survivors

^a^17 blood samples were missing at day 2 (9 patients died before day 2, 8 sample were missing)

^b^19 DNA samples were missing (3 patients died before DNA sample was obtained, and DNA sample was missing for 16 patients)

In our cohort of 261 genotyped patients, carrying *EPCR* haplotype A3 was associated with higher sEPCR at D1. Patients carrying a single A3 allele displayed elevated sEPCR (mean 159.9 ± 69.52 ng/mL) compared to non-A3 (81.25 ± 43.77 ng/mL), *p* < 0.001. Two patients homozygous (A3^+^/A3^+^) for the *EPCR* A3 allele had an average sEPCR level of 165.3 ± 60.20 ng/mL similar to patients who were heterozygous carrying a single A3 allele. However, no statistically significant difference in *EPCR* A3 allele carriage was observed between survivors and non-survivors (12.8% vs 15.5% respectively, *p* = 0.5804). Thus, carrying the *EPCR A3* allele did not constitute a significant predictive factor for survival in our study. Moreover, no interaction between *EPCR* genotype and the association between sEPCR plasma level and septic patient outcome was significant (*p* > 0.05). These results suggest no apparent impact of the *EPCR* genotype. According to these data, the sEPCR plasma level could represent a possible prognostic factor of in-hospital patient survival.

### Weight of sEPCR levels in multivariate analysis and survival prognostic scoring system

First, a multivariate analysis was performed. This analysis highlighted the following parameters as risk factors: age, gender, McCabe score, Fine’s score, SOFA, SAPS2, sEPCR level at D1 and variation in sEPCR values between D2 and D1 (Table 3). Our findings indicate a significant association between high level of sEPCR at D1 and an increased risk of mortality (OR = 1.95; *p* = 0.0407). Moreover, the increase in sEPCR from D1 to D2 was also associated with an increased risk of death (OR = 1.01; *p* = 0.0323).

**Table 3:** Multivariate analysis of the in-hospital mortality and prognostic scoring system at day 2 (*n* = 248)

Prognostic markers	Logistic regression			Scoring system
	Odds ratio	*P* value	95% CI	Weight
sEPCR log at day 1 (ng/mL)	1.95	0.0407	[1.03–3.70]	0.289
delta sEPCR (day 2 - day 1, ng/mL)	1.01	0.0323	[1.00–1.03]	0.269
Age (years)	1.03	0.0586	[1.00–1.05]	0.293
McCabe score at day 1	0.52	0.1833	[0.19–1.37]	−0.014
Gender, male versus female	2.02	0.0586	[0.97–4.19]	0.384
Fine’s score (log)	2.94	0.1928	[0.73–11.8]	0.341
SOFA score at day 1 (square)	1.01	0.0199	[1.00–1.01]	0.441
SAPS II	1.02	0.2136	[0.99–1.04]	0.257

The scoring system and the multivariate analysis were based on least absolute shrinkage and selection operator (lasso) logistic regression. The prognostic score was the sum of the product between the weights and the normalized explicative variables

*sEPCR* soluble endothelial protein C receptor, *SOFA* Sepsis-related Organ Failure Assessment, *SAPS* Simplified Acute Physiology Score

**Fig. 2:**
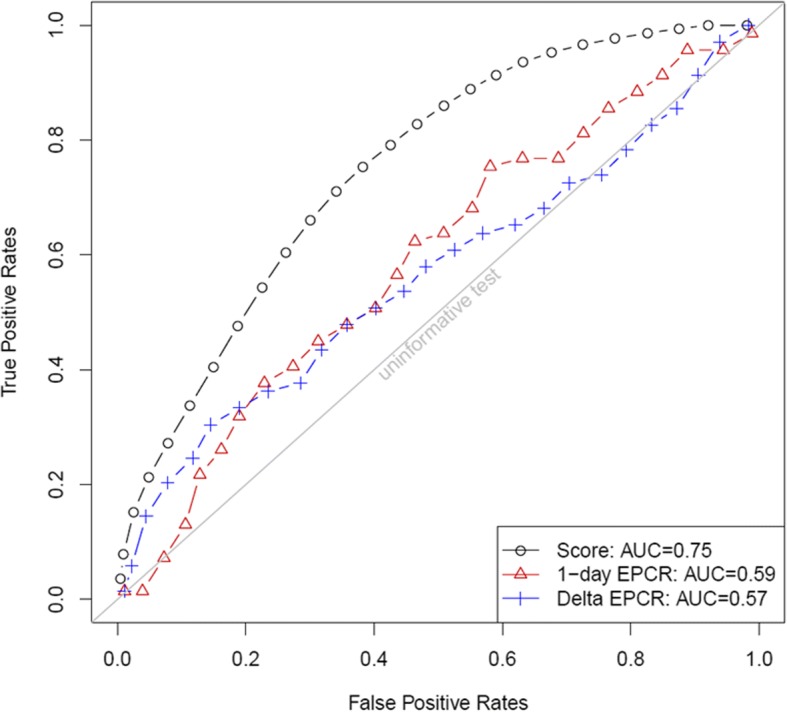
ROC curve for the scoring system at day 2 (SD-2). The 0.632+ bootstrap estimator was used to evaluate the scoring system SD-2 and to produce the corresponding ROC curve. The AUC obtained was 0.75. The soluble endothelial protein C receptor (sEPCR) level at day 1 and its time course from day 1 to day 2 were associated with univariate AUC values of 0.59 and 0.57, respectively

Next, to further evaluate the prognostic capacity of sEPCR we used a mortality scoring system that we previously described (22). In this scoring system, when the weight is positive (OR > 1), scoring system increases with the value of this risk factor (Table 3). Parameters of the scoring model at D2 (SD-2) for predicting mortality of a patient with septic shock are detailed in Additional file 1. Figure 2 shows the area under ROC curve (AUC) associated with the score (AUC = 0.75). In comparison, sEPCR level at D1 and its early time course from D1 to D2 taken as single predictors were associated with an AUC of 0.59 and 0.57, respectively, thus indicating a limited predictive value.

## Discussion

In several studies, sEPCR levels in septic patients were found to be significantly higher [11, 15], similar to [24–29] or even lower [25] than in healthy or non-septic controls. A possible explanation for these discrepancies is the bimodal distribution of sEPCR levels related to the A3 haplotype, as this gene polymorphism was associated with high levels of sEPCR. In a preliminary study, we compared the sEPCR levels at the time of admission (D1) between patients with severe sepsis and healthy patients and we found no significant difference [18]. However, that study was based on a small cohort of patients and the impact of the *EPCR* genotype was not examined. Additionally, we reported an early, transient, but significant rise in sEPCR levels at D2 in patients who did not survive [18]. In addition, Vassiliou et al. showed that the level of sEPCR at ICU admission was higher in patients who were originally non-septic who subsequently became septic compared to those who did not [28]. Consequently, these data suggested that sEPCR level and its temporal change could provide an early biological marker of sepsis outcome.

This observational study was designed to investigate further the prognostic value of sEPCR in patients with septic shock and pneumonia. For this purpose, a larger and homogenous cohort of independent patients (compared to our preliminary study) was analysed. A major result was the higher sEPCR level at D1 in non-survivors versus survivors at hospital discharge. This elevated baseline level of sEPCR was not the result of different incidence of *EPCR A3* haplotype. We focused our analysis on the early phase of septic shock (the first 24 h of care) by performing a dynamic analysis of the sEPCR levels. Indeed, sepsis is a dynamic process that evolves extremely fast, especially in the most severe forms. A kinetics analysis rather than a static analysis thus had pathophysiological and clinical meaning. The results are consistent with this view. A moderate elevation of around 4.4% in the sEPCR level from D1 to D2 was observed in patients who subsequently died. In contrast, a decrease of 4.0% was observed in patients who survived. Together, these data confirmed the EPCR pathway as a key biological factor in severe sepsis. Recent studies reported on parasites (*Plasmodium falciparum*) from patients with severe malaria that preferentially bind to EPCR [29, 30]. These studies represent a breakthrough in malaria pathogenesis research because they provide a link between pathophysiological mechanisms and parasite cyto-adhesion. These data suggest that malaria-associated loss of EPCR combined with parasite impairment of the EPCR–APC interaction may promote coagulation, inflammation, and endothelial barrier breakdown. Interestingly, soluble EPCR shed from endothelial cells by treatment with the metalloproteinase TNF-α converting enzyme was shown to block parasite attachment to the vessel wall cells. Ultimately, these researchers also propose the development of new strategies to disrupt the parasite–EPCR interaction or counteract its effects [31]. Whether similar mechanisms may occur in sepsis is still unknown.

We also studied the prognostic value of the sEPCR level at D1 and its course between D1 and D2, independently of the most relevant characteristics and scoring systems reflecting patient health state at baseline, i.e. SOFA, McCabe score, SAPS II, age, and Fine’s score. In other words, the sEPCR level at D1 and its course between D1 and D2 might constitute additional information for understanding mortality.

Our study confirms that *EPCR* A3 haplotype is strongly associated with sEPCR level in septic patients, consistent with the recent data reported by Vassiliou et al. [32]. However, we found no relationship between the *EPCR* gene polymorphism and sepsis prognosis. The genetic polymorphism of *EPCR* is the primary factor known to influence the basal levels of sEPCR [16, 32]. In this cohort the prevalence of *EPCR* A3 allele was 15%. These data are consistent with the prevalence described in the general population [16] and with our previous findings in a cohort of transplant donors [26]. A first study in the ICU setting showed an under-representation of the *EPCR* A3 allele in patients hospitalized for sepsis [27], suggesting the need to further investigate the possible involvement of the *EPCR* gene variant in the prognosis of sepsis. Vassiliou et al*.* recently found, consistent with our findings, that the distribution of the A3 allele was similar among survivors and non-survivors [32].

As usual for such an observational study, our study had several limitations that must be acknowledged. First, causality cannot be interpreted: soluble EPCR level and variation of EPCR are significantly associated with mortality risk, but the direct implication of EPCR and its release as a pathogenic factor remains to be established. Nevertheless, these results suggest that in septic shock, an early anomaly in the regulation of plasma levels of EPCRs although moderate, contrasting with the stability of sEPCR levels, is associated with poor outcome. Second, even if we elaborated an adjusted model taking confounders into consideration, we could not exclude non-observed confounding factors. Third, even if we used a statistical method for an internal validation of its prognostic capacities (AUC = 0.75), the scoring system we proposed has to be externally validated. There was also no power analysis, since the cohort was predefined before the study. Therefore, the biological pathway of sEPCR has yet to be studied to provide a biological marker for use in current clinical practice.

## Conclusions

Soluble EPCR level at baseline and its early increase were both significantly associated with in-hospital mortality in a large ICU cohort of patients treated for pneumococcal pneumonia with septic shock, although the *EPCR* genetic polymorphism was not associated with prognosis in septic patients. This study suggests that the sEPCR pathway could help us understand poor outcome in sepsis and thus suggests sEPCR as a key player in the pathogenesis of sepsis and as a potential biomarker of sepsis outcome. This last point has to be confirmed by external validation studies. In addition, the biological background of this early dysregulation in the release of sEPCR in sepsis still remains to be explored.

## Additional file

Additional file 1: The scoring system and the prognostic score at day 2 to predict in-hospital mortality. The scoring system and the multivariate analysis were based on lasso logistic regression. The prognostic score was the sum of the product between the weights and the normalized explicative variables. (DOCX 26 kb)

## Abbreviations

APC: Activated protein C
AUC: Area under the curve
D1: Day 1
D2: Day 2
ELISA: Enzyme-linked immunosorbent assay
EPCR: Endothelial protein C receptor
ICU: Intensive care unit
lasso: Least absolute shrinkage and selection operator
mEPCR: Membrane endothelial protein C receptor
OR: Odds ratio
PC: Protein C
ROC: Receiver operating characteristic
SAPS: Simplified Acute Physiology Score
SD: Standard deviation
SD-2: Scoring system at day 2
sEPCR: Soluble endothelial protein C receptor
SOFA: Sequential Organ Failure Assessment
TM: Thrombomodulin
TNF: Tumour necrosis factor
