# Supplementary material for: An early increase in endothelial protein C receptor is associated with excess mortality in pneumococcal pneumonia with septic shock in the ICU
Source: Crit Care. 2018 Oct 5;22:251. doi: 10.1186/s13054-018-2179-6 (PMC6173894; doi:10.1186/s13054-018-2179-6)
Supplement: Supplementary file 1 — The scoring system and the prognostic score at day 2 to predict in-hospital mortality. The scoring system and the multivariate analysis were based on lasso logistic regression. The prognostic score was the sum of the product between the weights and the normalized explicative variables. (DOCX 26 kb) [file 13054_2018_2179_MOESM1_ESM.docx]

Additional file 1: The scoring system to predict the in-hospital mortality after the day 2

The scoring system and the multivariate analysis were based on a Lasso logistic regression. In the scoring system, when the weight is positive (OR>1), SD-2 increases with the value of this risk factor. For instance, SD-2 increases with the patient age, the Fine’s score, the SOFA, the SAPS II and the EPCR level. SD-2 is also more important for men. In contrast, if the weight is negative (OR<1), SD-2 decreases for higher values. The score is lower for patients with a Mc Cabe’s scale equals 1.

|  | **Scoring System at Day 2** | | |
| --- | --- | --- | --- |
| **Explicative variables** | **Mean** | **SD** | **Weight** |
| **sEPCR log at day 1 (ng/ml)** | 4.365 | 0.565 | 0.289 |
| **delta sEPCR (day 2-day 1, ng/ml)** | -1.283 | 24.410 | 0.269 |
| **Age (years)** | 64.363 | 15.481 | 0.293 |
| **Score de Mac Cabe à 1** |  |  | -0.014 |
| **Gender, Male vs Female** |  |  | 0.384 |
| **FINE’s score (log)** | 4.927 | 0.329 | 0.341 |
| **SOFA’s score at day 1 (square)** | 109.888 | 78.602 | 0.441 |
| **SAPS II** | 52.932 | 17.716 | 0.257 |

The prognostic score was the sum of the product between the weights and the normalized explicative variables (subtraction by their mean and division by the corresponding standard deviation).

**SD-2 = 0.384 (if male, 0 if female) - 0.014 (if the Mc Cabe’s score is 1, 0 otherwise) + 0.293 (age in years - 64.363)/15.481 + 0.341 (logarithm of the Fine’s score - 4.921)/0.329 + 0.441 (SOFA2 - 109.888)/78.602 + 0.257 (SAPSII - 52.932)/17.716 + 0.289 (logarithm of the EPCR at day 1 in ng/ml - 4.365)/0.565 + 0.269 (EPCR at day 2 - EPCR at day 1 in ng/ml +1.283)/24.410**
